# Emotional Regulation in Young Adults with Internet Gaming Disorder

**DOI:** 10.3390/ijerph15010030

**Published:** 2017-12-25

**Authors:** Ju-Yu Yen, Yi-Chun Yeh, Peng-Wei Wang, Tai-Ling Liu, Yun-Yu Chen, Chih-Hung Ko

**Affiliations:** 1 Department of Psychiatry, Faculty of Medicine, College of Medicine, Kaohsiung Medical University, Kaohsiung City 807, Taiwan; yenjuyu@cc.kmu.edu.tw (J.-Y.Y.); y7552156@gmail.com (Y.-C.Y.); wistar.huang@gmail.com (P.-W.W.); li52030tw@gmail.com (Y.-Y.C.); 2 Department of Psychiatry, Kaohsiung Municipal Ta-Tung Hospital, Kaohsiung Medical University, Kaohsiung City 801, Taiwan; 3 Graduate Institute of Medicine, College of Medicine, Kaohsiung Medical University, Kaohsiung City 807, Taiwan; dai32155@gmail.com; 4 Department of Psychiatry, Kaohsiung Medical University Hospital, Kaohsiung Medical University, Kaohsiung City 807, Taiwan; 5 Department of Psychiatry, Kaohsiung Municipal Hsiao-Kang Hospital, Kaohsiung Medical University, Kaohsiung City 812, Taiwan

**Keywords:** Internet gaming disorder, IGD, emotional regulation, cognitive reappraisal, suppression, depression, hostility

## Abstract

People diagnosed with Internet gaming disorder (IGD) have been frequently reported to experience depression, anxiety, and hostility. Emotional regulation contributes to these mood symptoms. This study evaluated emotional regulation in subjects with IGD and examined relationships between emotional regulation, depression, anxiety, and hostility in young adults with IGD. We recruited 87 people with IGD and a control group of 87 people without a history of IGD. All participants underwent a diagnostic interview based on the IGD criteria of the Diagnostic and Statistical Manual of Mental Disorders, Fifth Edition, and they completed a questionnaire on emotional regulation, depression, anxiety, and hostility. We found that subjects with IGD were less likely to practice cognitive reappraisal and were more likely to suppress their emotions. Linear regression revealed the higher cognitive reappraisal and lower expressive suppression associated with depression, anxiety, and hostility among subjects with IGD. The emotional regulation strategies that characterize those with IGD could be contributing factors to the depression and hostility tendencies of these people. When treating patients with IGD, in addition to providing appropriate interventions to relieve depression and hostility, practitioners should effectively assess emotional regulation strategies and provide emotional regulation therapy to prevent a vicious cycle of negative emotions.

## 1. Introduction

Diagnostic criteria for Internet gaming disorder (IGD), defined as an addiction to Internet games, are proposed as research criteria in section III of the Diagnostic and Statistical Manual of Mental Disorders, Fifth Edition (DSM-5) [1]. IGD is one type of internet addiction and has been associated with mood-related psychopathological symptoms, such as depression and irritability [2,3]. This comorbidity could contribute to treatment difficulties and a poor prognosis of addictive disorder [4], for example, the comorbidity of depression associated with the higher psychosocial burden among subjects with IGD [5]. Further, comorbidity could indicate a causal relationship between the disorders [6] or a common factors model [7], wherein a shared mechanism generally accounts for increased comorbidity. To intervene, the shared mechanism might benefit both disorders. Therefore, understanding the shared mechanism causing comorbidity between IGD and psychopathological symptoms could contribute to successfully developing treatments for them.

### 1.1. Association between IGD and Emotional Difficulties

The amount of time spent playing online games has been positively correlated with depressive symptoms [8,9]. The association between IGD, depression, and hostility were also demonstrated in recent studies [10,11]. Gentile et al. reported that IGD could be a cause of depression in adolescents [12]. Further, Ciarrochi et al. also reported that compulsive internet use predicted poor mental health among adolescents in longitudinal investigation [13]. These results might indicate that repeatedly excessive online gaming could contribute to emotional difficulties, possibly though impaired daily life functions or their negative consequences. On the other side, addictive behavior, such as online gaming [14], could be a way of coping with pre-existing emotional difficulties, such as depression [6]. Depression was reported to predict the incidence of internet addiction and support this claim [15]. This might suggest that emotional difficulties could possibly contribute to IGD; however, this has not been proved. The possible bidirectional effect between IGD and emotional difficulties deserves future prospective study. On the other hand, an underlying factor, such as emotional regulation, might be associated with both IGD and emotional difficulties, and could contribute to the comorbidity of IGD.

### 1.2. Emotional Regulation and Depression, Anxiety, Hostility, and IGD

Emotional regulation, also known as emotional self-regulation, was defined by [16] as the set of cognitive processes that influence emotional responses. Emotional regulation is a complex process that includes the initiation, inhibition, or modulation of aspects of emotion functioning. A previous review demonstrated that interventions that specifically target emotional regulation can not only promote positive emotional regulation but also attenuate associated psychopathological symptoms [17].

Two strategies are commonly used for downregulating emotion. The first, reappraisal, comes early in the emotion-generative process and entails changing how a situation is construed in order to reduce its emotional impact. The second, suppression, comes later in the emotion-generative process and entails the inhibition of outward signs of inner feelings [18]. The two types of emotional regulation are evaluated in The Emotional Regulation Questionnaire, which measures the habitual use of expressive suppression and cognitive re-evaluation. The scale includes items related to the regulation of positive and negative emotions [19]. According to this measurement, practicing reappraisal is associated with greater positive emotion, improved interpersonal functioning, and well-being. By contrast, practicing suppression is associated with negative emotions and poorer interpersonal functioning. These results suggest that strategies that act early in the emotion-generative process have a different profile of consequences than strategies that act later.

Emotional regulation was associated with depression [20] and anxiety [21]. The employment of adaptive emotional regulation strategies (e.g., reappraisal) causes a reduction in stress-induced emotions. Conversely, dysfunctional emotional regulation strategies, such as emotion suppression, appear to influence the pathogenesis of depression. For example, a structural equation modeling study found that expressive suppression mediated the relationship between intensity of negative affect and psychological distress [22]. In addition, emotional regulation therapy has been reported to be an effective treatment of emotional dysfunctions, such as anxiety or depression [17,23,24]. The literature demonstrates the role for emotional regulation in the development or maintenance of depression and anxiety [20,21].

Fewer studies have evaluated the relationship between emotional regulation and hostility than the relationship between emotional regulation and depression or anxiety. People with lower anger control can reasonably be assumed to display more aggressive behaviors [25]. A previous study demonstrated the relationship between emotional regulation and anger reactivity [26]. Hostile cognition is a major factor contributing to anger and aggressive behavior [27]. However, whether cognitive appraisal can attenuate the role of hostile cognition in depression has not been evaluated.

Depression and emotional regulation are considered risk factors for the development of addictive disorders [28]. Emotional regulation was reported to predict substance use disorder (specifically, alcohol use disorder [29]) and has been suggested to have a moderating role in addiction development [30]. IGD has been reported to be associated with depression, irritability, and anxiety [2,3,31]. Difficulties with emotional regulation are associated with these associated psychopathological symptoms [20,21]. Furthermore, poor emotional regulation might contribute to depression [20] that predicts IGD [15,32]. Moreover, excessive online gaming could have negative consequences that could result in stress for individuals with IGD. Appropriate emotional regulation mediates negative effects and psychological stress [22], whereas impaired emotional regulation might contribute to mood symptoms, such as depression and anxiety. Loton et al. revealed that the coping strategy had been reported to account for the association between video gaming addiction and depression [14]. It supported the claim that inappropriate emotional regulation might contribute to the association between psychopathological symptoms of IGD. However, the association between emotional regulation and these psychopathological symptoms has not been evaluated among subjects with IGD.

### 1.3. Study Hypothesis and Objectives

We hypothesized that emotional regulation, cognitive reappraisal, and suppression are associated with IGD, and that individuals with IGD practice less emotional regulation, use fewer reappraisal strategies, and tend to suppress emotions more than does the average person. Furthermore, the deficit in emotional regulation might be correlated with depression, hostility, and anxiety among subjects with IGD. Accordingly, this study evaluated the following: (1) cognitive reappraisal and expressive suppression among individuals with and without IGD, and (2) the associations among cognitive reappraisal, expressive suppression, depression, hostility, and anxiety among subjects with IGD.

## 2. Materials and Methods

### 2.1. Participants

Our participants, namely individuals with current IGD (the IGD group) and those with no history of IGD (the control group), were recruited through advertisements that demonstrated our recruitment critieria on campuses and bulletin board systems at universities in Taiwan between September 2012 and October 2013. Our recruitment criteria for the IGD group, which were based on an fMRI study for young adults with IGD, were as follows [32]: (1) aged 20–30 years with education of >9 years; (2) played Internet games for ≥4 h per day on weekdays and ≥8 h per day on weekends or for ≥40 h per week; and (3) had maintained an Internet gaming pattern for >2 years. The recruited participants spent most of their free time on Internet gaming. For participants fulfilling these criteria, a psychiatrist conducted an interview, during which the DSM-5 diagnostic criteria for IGD was used [1] in the interviewing room at laboratory. Participants that fulfilled the DSM-5 criteria of IGD were classified in the IGD group.

For every participant enrolled in the IGD group, a gender-, age- (within a range of 1 year), and education level-matched control participant was recruited according to the criteria that their nonessential Internet use was of <4 h per day in their daily life. The limitation on internet use was designed to prevent recruiting subjects with internet addiction in control group. Then, these participants also underwent a diagnostic interview with the psychiatrist based on DSM-5 criteria of IGD to confirm their recruitment in control group.

The diagnostic interview comprised two parts: (1) a diagnostic interview based on the Chinese version of the Mini-International Neuropsychiatric Interview (MINI) to reveal existing psychotic disorders, bipolar I disorder, and substance use disorders; and (2) a history-taking interview to determine psychotropic medication use, mental retardation, severe physical disorder, and brain injury. Individuals with psychotic disorders, bipolar I disorder, substance use disorders, psychotropic medication use, mental retardation, severe physical disorder, or brain injury were excluded. In total, 174 participants—87 in each group—were included after diagnostic interviewing and their informed consent was obtained. Then, study participants completed the assessment in this present study. This study was approved by the Institutional Review Board of Kaohsiung Medical University Hospital.

### 2.2. Measures

DSM-5 diagnostic criteria for IGD [1]. The DSM-5 IGD diagnostic criteria include comprises nine items: preoccupation, withdrawal, tolerance, unsuccessful attempts to control, loss or decrease of other interests, continued excessive use despite psychosocial problems, deceiving, escapism, and functional impairment [1]. We developed a semistructured interview for examining the DSM-5 criteria for IGD. Participants fulfilling ≥5 criteria were included in classified as the IGD group.

Chinese version of the MINI [33]. We conducted a diagnostic interview to rule out psychiatric disorders by using the modules of psychotic disorders, bipolar I disorder, and substance use disorders in the Chinese version of the MINI. Those with existing disorders were excluded from the study.

Emotional regulation questionnaire. The emotional regulation questionnaire (ERQ) is a 10-item scale designed to measure respondents’ tendency to regulate their emotions in two ways: (1) cognitive reappraisal, assessed using a reappraisal scale (six items such as “When I want to feel less negative emotion (such as sadness or anger), I change what I’m thinking about”), and (2) expressive suppression, assessed using a suppression scale (four items such as “I control my emotions by not expressing them”). Respondents answer each item on a 7-point Likert-type scale from 1 (strongly disagree) to 7 (strongly agree). The alpha reliabilities were averaged 0.79 and 0.73 for the reappraisal and suppression scales, respectively. Test–retest reliability over 3 months was 0.69 for both scales in its original study [19]. There are several scales assessing emotional regulation. We utilized ERQ to assess the most important two strategy of emotional regulation because of its brief and convenient nature.

Depression, hostility, and anxiety were assessed by the Center for Epidemiological Studies’ Depression Scale (CES-D) [34,35] Penn State Worry Questionnaire (PSWQ) [36] and the Buss–Durkee Hostility Inventory Chinese Version—Short Form (BDHIC-SF) [37]. Cronbach’s alpha of CES-D, PSWQ, and BDHIC-SF in the present study were 0.92, 0.90, and 0.92, respectively. Higher score of CES-D, BDHIC-SF, and PSWQ indicates higher depression, hostility, and anxiety, respectively.

### 2.3. Statistical Analysis

We first evaluated the differences in cognitive reappraisal and expressive suppression between the IGD and control groups. Logistic regression was used to regress the diagnosis of IGD on the reappraisal and suppression while controlling for gender, age, and educational level. Then, linear regression was used to regress the depression on the cognitive reappraisal, and expressive suppression with control of gender, age, and educational level in both IGD and control group. The gender was set as female = 0 and male = 1 in the linear regression. The same method was used to evaluate the associations between reappraisal, suppression, and hostility or anxiety. *p* < 0.05 was considered significant in the analyses, all of which were performed using SPSS. The significant threshold of multiplicity was corrected using the Holm–Bonferroni methods. The Holm–Bonferroni method controls the familywise error rate (Type I errors) by adjusting the *p* value of the individual comparison [38].

## 3. Results

### 3.1. Gender, Age, and Education Levels

Eighty-seven people were recruited for each group. Their gender (X^2^ = 0, *p* = 1), age (*t* = 0.26, *p* = 0.80), and education levels (*t* = 1.15, *p* = 0.25) did not differ significantly (Table 1).

### 3.2. Emotional Regulation and IGD

The IGD group had significantly lower cognitive reappraisal strategies (*t* = −2.64, *p* = 0.009) and greater expressive suppression strategies (*t* = 2.29, *p* = 0.02) than did the control group (Table 1). Logistic regression (Table 2) revealed that cognitive reappraisal negatively predicts IGD (odds ratio; OR = 0.91; 95% CI = 0.85–0.97) and that expressive suppression positively predicts IGD (OR = 1.14; 95% CI = 1.04–1.25).

### 3.3. Within-Group Analysis for Emotional Regulation 

Multiple linear regression analysis was used to test if the emotional regulation significantly predicted depression, anxiety, or hostility of subjects in IGD group (Table 3). The results indicated the model explained 19% of the variance in depression (*R*^2^ = 0.19, *F*_(5,81)_ = 3.74). Cognitive reappraisal significant predicted depression (*B* = −0.72, *t* = −3.66, *p* < 0.001), as did expressive suppression (*B* = 1.02, *t* = 3.24, *p* = 0.002). Further, the model explained 18% of variance in anxiety (R^2^ = 0.18, *F*_(5,81)_ = 3.59). Cognitive reappraisal significant predicted anxiety (*B* = −0.69, *t* = −3.20, *p* = 0.002), as did expressive suppression (*B* = 0.91, *t* = 2.66, *p* = 0.01). The model also explained 12% of variance in hostility (*R*^2^ = 0.12, *F*_(5,81)_ = 2.2). Cognitive reappraisal significantly predicted hostility (*B* = −0.75, *t* = −2.79, *p* = 0.007), as did expressive suppression (*B* = 1.09, *t* = 2.53, *p* = 0.01). These results suggested that IGD subjects with lower cognitive reappraisal and higher expressive suppression had higher depression, anxiety, and hostility. We also provide the result in control group. It demonstrated the similar association between emotional regulation and depression, anxiety, and hostility in control group (Table 3).

## 4. Discussion

People with poor emotional regulation often engage in maladaptive behavior to escape from their emotions, creating risks of a range of mood disorders and addictive disorders [39]. Thus, such people have been associated with various addictive disorders [29,30]. To our knowledge, no previous study has assessed emotional regulation among subjects with IGD. As expected, the present study demonstrated that subjects with IGD have lower cognitive reappraisal and higher expressive suppression. This result is similar to a previous report demonstrating lower cognitive reappraisal in gambling disorder [39]. Further, our study demonstrated that lower cognitive reappraisal and higher expressive suppression were associated with depression, anxiety, and hostility among subjects with IGD.

Our literature review suggested that those individuals experiencing depression or anxiety have ineffective emotional regulation and difficulties in processing negative emotions [20,21]. Cognitive reappraisal is a cognitively-oriented strategy for redefining emotional stimuli in unemotional terms or for reimagining depressive situations [40]. It comes early in the emotion-generative process and effectively decreases the experience of negative emotions [18]. By contrast, expressive suppression, coming later in the emotion-generative process, entails the inhibition of outward signs of inner feelings. Suppression is ineffective for down-regulating negative emotions, and people with a history of depression have been reported to spontaneously use this strategy [41]. Like these previous results, our results demonstrated that subjects with higher depression have lower cognitive reappraisal and higher expressive suppression among both subjects with IGD and controls.

People with IGD experience negative psychosocial consequences from excessive online gaming [42]. They also experience depression, anxiety, or irritation when they are prohibited from playing games online [1]. Thus, previous prospective study had suggested that internet gaming disorder or excessive online gaming [8,12] contributes to depression. They could reappraisal that this is a logical result of ceasing an excessive, self-gratifying behavior, and that the depression and restlessness could be avoided if they engaged in an alternative, appropriate activity such as exercise. However, without appropriate reappraisal, subjects with IGD could experience depression. Further, continuing to suppress negative emotions rather than reappraising them could leave these emotional difficulties unresolved. Thus, the lower cognitive reappraisal and higher suppression of subjects with IGD could partly account for their vulnerability to depression.

Although there is no report demonstrating the predictive effect of depression on internet gaming disorder, previous reports had suggested that depression predicted incidence of internet addiction [32]. Subjects with lower cognitive reappraisal that were habituated to use suppression could experience depression under stress [20,22]. Online gaming could provide a virtual world for people to escape from their negative emotions [43] and could buffer stress [44]. However, if the gaming time could not be well controlled, the repeatedly excessive gaming could result in further negative consequences among vulnerable subjects. It could create a vicious cycle and lead to repeated engagement in online gaming, resulting in increased addiction risk. Anyway, this claim should be further evaluated in prospective study.

Subjects with higher anxiety were more likely to pay attention to threat-related stimuli rather than neutral stimuli [45]. The continued attention to threat increases their cognitive and emotional response, contributing to anxiety symptoms. The manner in which information was processed in emotional regulation could determine anxiety severity [24]. The use of suppression as a regulatory mechanism and limited access to emotional regulation strategies, such as cognitive reappraisal, were associated with anxiety [46]. Thus, dysfunctional emotional regulation contributes to the development of anxiety disorder [24]. In this study, the anxiety of subjects with IGD is negatively associated with cognitive reappraisal and positively associated with expressive suppression.

In addition, reappraisal facilitates the adaptive processing of anger-inducing situations and contributes to anger regulation [47]. However, the suppression of anger could increase hostility under stress [48]. As expected, subjects with IGD habitually suppress emotions, or those unlikely to reappraise their negative cognition exhibited higher levels of hostility in this study. Moreover, suppression of hostility could increase sympathetic activity [49], as well as the risk of cardiovascular disorder [50]. Thus, emotional suppression and hostility of subjects with IGD might result in not only emotional difficulty but also cardiovascular risk.

Cognitive control ability is essential and contributes to emotional regulation, such as reappraisal [40]. Subjects with IGD had impaired cognitive control [51], similar to people with gambling disorders [52] and addictive disorder, such as cocaine use disorder [53]. The impaired cognitive control ability could associate with their impaired cognitive reappraisal in subjects with IGD. Further study is necessary to understand the neurocognitive mechanism of the impaired emotional regulation, such as cognitive control, among subjects with IGD.

### 4.1. Clinical Implication

The dysfunctional emotional regulation of subjects with IGD was associated with depression, anxiety, and hostility [32]. Emotional regulation should be well assessed and intervened in among young adults with IGD. Three key steps—emotional awareness, emotional regulation, and exchanging one emotion for another—help people to modify the state, the belief, and the behavior in response to emotion-eliciting events. These interventions for emotional regulation [23] have been recommended for the treatment of depression [20]. Evidence-based emotion management strategies, such as emotion-focused therapy [54], could be provided to young adults with IGD to promote cognitive reappraisal and attenuate expressive suppression strategies and responses. They must become aware that their negative emotions result from the negative consequences of gaming or from the conflicts in their lives. Alternative activities, physical exercise, and further psychological support should be offered to help relieve negative emotions. Furthermore, information and guidance on reappraisal should be provided so that positive thinking can replace negative thinking. This intervention to promote reappraisal and prevent suppression may attenuate their depression, anxiety, and hostility, and prevent the vicious cycle of IGD. However, these claims for the effects of emotional regulation therapy should be evaluated with future clinical research.

### 4.2. Limitations

This study has three limitations. First, emotional regulation was assessed using a questionnaire only and not through the investigation of real situations. Second, IGD was diagnosed only through diagnostic interviews with the participants, and supplementary information from family members or partners, which could have contributed to verifying the validity of the diagnoses, was not collected. Third, our cross-sectional research design could not confirm causal relationships between emotional regulation and IGD. Besides, the structure equation model had not been utilized to test hypothesized model because of unconfirmed causal relationship.

## 5. Conclusions

People with IGD practice less cognitive reappraisal and more suppression. In this study, people practicing less cognitive reappraisal and more suppression had more symptoms of depression, anxiety, and hostility, suggesting that impaired emotional regulation might exacerbate negative mood symptoms in people with IGD. Thus, emotional regulation should be effectively assessed when treating people with IGD. Furthermore, this group should be given interventions to promote cognitive reappraisal and attenuate expressive suppression in order to avoid a vicious cycle of negative emotions.

## Figures and Tables

**Table 1:** Age, educational level, emotional regulation, hostility, depression, and severity for the IGD and control groups.

Variables	IGD Diagnosis		X^2^
	Yes (*N* = 87)	No (*N* = 87)	
	Mean ± SD	Mean ± SD	*t*-Test
Gender			
Male	70 (80.5%)	70 (80.5%)	0.00
Female	17 (19.5%)	17 (19.5%)	
Age	23.29 ± 2.34	23.38 ± 2.40	0.26
Education level	15.93 ± 1.15	16.14 ± 1.22	1.15
Cognitive reappraisal ^1^	31.09 ± 5.43	33.16 ± 4.87	−2.64 **
Expressive suppression ^2^	19.22 ± 3.40	17.98 ± 3.74	2.292 *

* *p* < 0.05; ** *p* < 0.01; ^1^ Score of cognitive reappraisal subscale of *ERQ*; ^2^ Score of expressive suppression subscale of *ERQ*.

**Table 2:** Logistic regression to evaluate the predictive value of emotional regulation in IGD with control of gender, age, and educational level.

Variables	Wald	Exp(β)	95% CI
Among all subjects			
Gender	0.01	1.05	0.47–2.32
Age (year)	0.43	1.0550	0.91–1.22
Education level (year)	1.11	0.86	0.64–1.14
Cognitive reappraisal ^1^	8.97 **	0.91	0.85–0.97
Expressive Suppression ^2^	7.28 **	1.14	1.04–1.25

** *p* < 0.01; ^1^ Score of cognitive reappraisal subscale of *ERQ*; ^2^ Score of expressive suppression subscale of *ERQ*.

**Table 3:** Multiple linear regression analysis for the predictive value of emotional regulation in depression, hostility, and CGI score among IGD group or control group.

Variables	*B*	IGD	*p*	*B*	Control	*p*
		*t*			*t*	
Depression ^1^						
Gender	0.78	0.31	0.76	−1.76	−1.06	0.29
Age (year)	0.24	0.52	0.61	−0.24	−0.77	0.44
Education level (year)	−0.19	−0.20	0.84	0.21	0.34	0.73
Cognitive reappraisal ^2^	−0.72	−3.66	<0.001	−0.73	−5.21	<0.001
Suppression ^3^	1.02	3.24	0.002	1.06	6.01	<0.001
	*F*_(5,81)_ = 3.74	*R*^2^ = 0.19		*F*_(5,81)_ = 12.90	*R*^2^ = 0.44	
Anxiety ^4^						
Gender	−1.86	−0.68	0.50	−2.75	−1.29	0.20
Age (year)	−0.79	−1.57	0.12	−0.72	−1.78	0.08
Education level (year)	1.39	1.34	0.19	1.58	1.98	0.05
Cognitive reappraisal	−0.69	−3.20	0.002	−0.96	−5.28	<0.001
Suppression	0.91	2.66	0.01	1.13	4.93	<0.001
	*F*_(5,81)_ = 3.59	*R*^2^ = 0.18		*F*_(5,81)_ = 11.71	*R*^2^ = 0.42	
Hostility ^5^						
Gender	−1.19	−0.34	0.73	−3.61	−1.52	0.13
Age (year)	0.13	0.20	0.84	0.09	0.21	0.84
Education level (year)	−0.60	−0.46	0.65	0.15	0.17	0.87
Cognitive reappraisal	−0.75	−2.79	0.01	−0.72	−3.55	0.001
Suppression	1.09	2.53	0.01	1.45	5.69	<0.001
	*F*_(5,81)_ = 2.20	*R*^2^ = 0.12		*F*_(5,81)_ = 8.87	*R*^2^ = 0.35	

^1^ Score of cognitive reappraisal subscale of *ERQ*; ^2^ Score of expressive suppression subscale of *ERQ*; ^3^ Score of Center for Epidemiological Studies’ Depression Scale; ^4^ Score of PSWQ; ^5^ Score of the Buss–Durkee Hostility Inventory—Chinese Version—Short Form.
